# Efficacy and safety outcomes of the Paul glaucoma implant compared to the Ahmed glaucoma valve

**DOI:** 10.1038/s41598-025-00839-0

**Published:** 2025-05-13

**Authors:** Angi Lizbeth Mendoza-Moreira, Julia V. Stingl, Anna Maria Voigt, Jasmin Rezapour, Achim Fiess, Felix Mathias Wagner, Alexander K. Schuster, Esther M. Hoffmann

**Affiliations:** Department of Ophthalmology, University Medical Center Mainz, Langenbeckstrasse 1, 55131 Mainz, Germany

**Keywords:** Ahmed glaucoma valve, Drainage, Paul glaucoma implant, Glaucoma, Tube, Ocular hypertension, Glaucoma

## Abstract

This study compares the one-year outcomes of standalone Ahmed glaucoma valve (AGV) implantation and standalone Paul glaucoma implant (PGI) in adult patients with primary and secondary glaucoma. A retrospective, single-center, comparative study was conducted on adult patients who underwent standalone PGI and AGV at the University Medical Center Mainz. The primary outcome measures were the changes of IOP and the number of antiglaucoma eye medication at one year postoperatively. Secondary outcome measures included complete and qualified success rates, failure rates, visual acuity logMAR and the incidence of adverse events. A total of 24 adult patients were included in the AGV group and 28 in the PGI group. The median preoperative intraocular pressure decreased from 29.5mmHg (Interquartile range (IQR) 21–42) to 16.0mmHg (IQR 7–37) in the AGV group, and from 34.0 mmHg (IQR 13–56) to 16.0 (IQR 7–21) mmHg in the PGI group at the one-year follow-up. The median number of classes of intraocular pressure-lowering medications reduced from 3.5 to 0 in the AGV group, and from 3.0 to 0 in the PGI group. There were no statistically significant differences between the groups for any success criteria or failure. The AGV produced more encapsulation than the PGI, and the latter more tube exposures. Both the Ahmed Glaucoma Valve and the Paul Glaucoma Implant effectively reduce IOP and the number of antiglaucoma medications at one year with comparable safety profiles.

## Introduction

Glaucoma drainage devices (GDD) are widely employed for managing intraocular pressure, especially in refractory glaucoma^1^. After the introduction of the nonvalved Molteno implant in the 1970s, the most popular GDDs, namely the Ahmed Glaucoma valve (AGV) and the Baerveldt glaucoma implant (BGI), emerged in the early 1990s^2^. The AGV (New World Medical Inc., Rancho Cucamonga, California, USA) is a glaucoma valve equipped with a silicone elastomer membrane within the tube to mitigate postoperative hypotony^1,3^. The tube has an inner diameter of 0.305 mm and received FDA approval in 1993^1,3,4^. At the 7-year follow-up, 78.7% of patients with an AGV achieved an intraocular pressure (IOP) of 6 mmHg or greater and 21 mmHg or less and/or an IOP reduction of 20% or more relative to preoperative values^1^. The BGI (Johnson & Johnson Vision, Santa Ana, CA, USA) has an inner diameter of 0.320 mm but no resistance to flow^4,5^ The long-term success rate is approximately 63–78%, with variability in the definition of success, using an upper limit of IOP between 18 mmHg and 21 mmHg^6–9^. Although both BGI and AGV are effective in controlling the IOP on long term, BGI shows less tendency to failure. However, it has more postoperative complications, above all hypotony, compared to AGV^7,10,11^. Postoperative complications of both implants include hypotony and its associated consequences, diplopia, device exposure, corneal endothelial failure, and bleb encapsulation among others^1,2,5^.

With the advancement in knowledge and better understanding about fluid dynamics in intraocular surgery, new implants have emerged^2,4,12^. One of them is the Paul Glaucoma implant (PGI). The PGI (Advanced ophthalmic innovations PTE. LTD. Singapore) received Conformité Europeenne (CE) mark in Europe in 2018^13,14^. The tube has an inner diameter of 0.127 mm, which is approximately half that of the BGI, while maintaining the same plate area^2,4,14^. It is designed to reduce hypotony, tube exposure, and endothelial cell loss compared to other tube implants^4^. An intraluminal 6 − 0 Prolene suture prevents hypotony in the early postoperative period and allows for subsequent intraocular pressure (IOP) control through extraction during postoperative weeks 8–12^12–16^. As an alternative, the tube could be ligated using an absorbable suture^13,17,18^. The 3-year results of the PGI studies showed a good postoperative control with a good safety profile^12^. There is a limited number of studies comparing PGI with other GDD^16,18^. A retrospective study conducted on patients with secondary glaucoma due to silicone oil implantation comparing AGV with PGI revealed that both procedures demonstrated comparable surgical success^16^. Nevertheless, PGI tended to have less postoperative complications^16^. The goal of this study is to compare the one-year efficacy and safety profile between the Ahmed Valve and the Paul glaucoma implant on patients that underwent tube implant in the University Medical Centre Mainz, Germany.

## Materials and methods

### Inclusion criteria

Patients aged 18 years and older with Stage 3 or higher (severe rim loss with extensive RNFL damage) according to the Glaucoma Damage Staging System (GDSS) who underwent standalone Paul Glaucoma Implant surgery with at least 12 months of follow-up were compared to the most recent patients who received a standalone Ahmed Glaucoma Valve with a 12-month follow-up at the University Medical Center Mainz, Germany, between 2019 and 2023.

### Exclusion criteria

Patients younger than 18 years, who underwent a combined procedure, or do not have a minimum of 12 months follow-up.

### Surgical techniques

#### Ahmed glaucoma valve

Disinfection of the eye to be operated on with povidone-iodine, looping of the cornea with 7.0 silk. Creation of a limbal-based conjunctival flap involved making an incision of the conjunctiva approximately 10 mm posteriorly from its insertion at the limbus. The quadrant for the incision was selected based on the available space and the quality of the conjunctiva and sclera observed intraoperatively by the surgeon. Priming of the Ahmed implant, and insertion of the implant body. Application of Mitomycin C (MMC) 0.2 mg/ml in the posterior Tenon space over the Ahmed plate using an 8 × 8 mm sponge, application duration 1–5 min, rinsing with 30 ml BSS. A crescent blade was used to create a partial-thickness scleral flap, measuring 3 × 4 mm, with the base toward the limbus. Fixation of the Ahmed plate with a 6 − 0 prolene suture. Trimming the tube. Paracentesis at 10 o’lock with insertion of Healon. Marking the entry point for the tube on the sclera 2 mm behind the limbus with a blue pencil under the flap. Puncturing into the anterior chamber with a 22-gauge cannula, followed by inserting the trimmed tube into the anterior chamber. Closure of the scleral flap with four 10 − 0 nylon sutures. Closure of Tenon’s capsule with 8 − 0 Vicryl in interrupted sutures and closure of the conjunctiva (continuous) with 8 − 0 Vicryl. Adjusting the anterior chamber form and pressure with balanced salt solution. Closure of the paracenteses. Verifying the eye pressure by palpating the cornea with a cannula. Removal of the traction suture. Some viscoelastic is left in the anterior chamber. Subconjunctival injection of 4 mg Dexamethasone, application of ofloxacin ointment, bandage.

If Tutoplast^®^ Fascia lata (Tutogen Medical GmbH, Germany) or Tutopatch^®^ Bovine Pericardium (Tutogen Medical GmbH, Germany) were used, no scleral flap was created. The entry point for the tube was marked directly on the sclera, 2 mm behind the limbus. Placing the mesh material (Tutopatch^®^ or Tutoplast^®^) on the tube and fixing it with 10.0 nylon were done after the trimmed tube was inserted into the anterior chamber.

#### Paul glaucoma implant

The steps are similar to AGV implantation, with a few key differences: an intraluminal 6 − 0 Prolene suture was inserted into the tube, the Paul implant was positioned beneath the adjacent rectus muscles and secured with 8 − 0 Prolene, and a 26-gauge needle was used to puncture the anterior chamber for insertion.

#### Study measurements

Post-operative data were collected at specific time points: day 1, month 1 (± 1 week), months 3 and 6 (± 2 weeks), month 12 (± 1 month).

#### Outcome measurements

##### Primary outcome measurements

Postoperative changes in intraocular pressure and number of antiglaucoma medications at one-year follow-up for AGV and PGI. IOP was measured using an applanation tonometer. Baseline IOP was recorded under topical medications, and when applicable, with the use of oral acetazolamide. The number of patients using oral acetazolamide was also documented.

##### Secondary outcome measurements

Postoperative changes in visual acuity, measured in logMAR, were assessed at the one-year follow-up for both AGV and PGI groups. Visual acuity was initially recorded on a decimal scale using the standardized Snellen method. These records were then converted into the Logarithm of the Minimum Angle of Resolution (logMAR) using the formula -log10(decimal visual acuity). The following low vision transformations into logMAR were used: finger count = 2.0, hand motion = 2.7 logMAR, light perception = 3.7 logMAR, and no light perception = 4.7 logMAR^19^.

Surgical success at postoperative IOP targets at one-year follow-up was assessed as follows: target 1 is ≤ 21 mmHg with a reduction of ≥ 25% from baseline, target 2 is ≤ 18 mmHg with a reduction of ≥ 30% from baseline, and target 3 is ≤ 15 mmHg with a reduction of ≥ 40% from baseline. Complete success was defined as achieving the final IOP target without the need for treatment, while qualified success was considered when the target IOP was reached with the inclusion of treatment. Failure was defined as not achieving the IOP target at least twice in consecutive appointments, removal of the implant, loss of light perception vision, or the need for further surgical intervention to control IOP, such as open revisions or new glaucoma surgery. Healon injections to manage hypotony were not classified as failures if they were not accompanied by surgical revision. Similarly, needling and Prolene extraction were also not considered failures. The use of oral acetazolamide to control intraocular pressure at the end of the follow-up period was deemed a failure.

The postoperative regimen was the same for all groups: Floxal AT 4 times daily for one week, Dexa-EDO 6 times daily, reducing by one drop per week, Prednisolone ophthalmic ointment at night for one week, and artificial tears 6 times daily for one month.

##### Intraoperative and postoperative complications

Numerical hypotony (IOP < 6 mmHg with no associated complications), clinical hypotony (IOP < 6 mmHg associated with choroidal detachment and/or anterior chamber depth reduction), choroidal detachment, severe anterior chamber shallowing (with iris-corneal touch), hyphema, endophthalmitis, IOP spike (elevation of 10 mmHg from preoperative IOP), corneal decompensation (corneal edema persisting for over 4 weeks), persistent uveitis (SUN grade > 1 + cell in the anterior chamber persisting for 6 weeks), no light perception, leak, ptosis, diplopia, corneal Dellen, dysesthesia, iridodialysis, iris atrophy, device obstruction, device malposition, fibrous/epithelial ingrowth, device migration, and device exposure. An early postoperative complication was defined as one that occurred within the first 3 months after surgery. The need for ripcord removal in the PGI group was analysed; however, it was not considered a criterion for surgical failure.

### Statistical analysis

Statistical analysis was conducted using IBM SPSS Statistics version 23. Statistical significance was set at a p-value < 0.05. Data normality was assessed with the Kolmogorov-Smirnov and Shapiro-Wilk tests and presented as counts (percentages), means (standard deviations or 95% confidence intervals), or medians (interquartile ranges). The Chi-squared test was used for categorical variables with expected counts ≥ 5; otherwise, Fisher’s Exact Test or the Likelihood Ratio Test was employed. Binary logistic regression was used to assess the relationship between two variables and determine whether there was a difference in incidence between the two groups. Due to the small sample size and low number of events, bootstrapping was employed to improve the reliability of the results. The Mann-Whitney U test was used for non-parametric intergroup comparisons, while the Wilcoxon signed-rank test assessed intragroup differences. The paired and un-paired Student’s t-test was used for parametric comparisons. Patients undergoing additional glaucoma surgery, implant removal, or experiencing loss of light perception were excluded from the analysis of mean/median intraocular pressure and medication counts from those events onward. Kaplan-Meier analysis and the log-rank test were used to evaluate complete and qualified success at different IOP targets.

### Ethics declarations

Due to the retrospective nature of the study, The Landesärztekammer Rheinland-Pfalz Ethics Committee waived ethical approval. This study was conducted adhering to the ethical standards set forth in the Declaration of Helsinki. Informed consent was obtained from each patient before the surgery.

## Results

### Baseline characteristics of study population

A total of 63 records were analyzed in the PGI group, of which 10 were excluded for not meeting the age criteria, and 25 were excluded for not meeting the required follow-up duration. In the AGV group, 73 patients were analyzed, with 32 excluded due to age and 17 excluded for not meeting the required follow-up period. A total of 24 patients in the AGV group, and 28 patients in the PGI group were included. The mean age (± SD) was 48.3 ± 17.7 years in the AGV group and 47.6 ± 16.8 years in the PGI group. The characteristics of the study population are summarized in (Table 1). There were significant differences in ethnicity and the use of an intraoperative patch.

**Table 1:** Baseline demographic and clinic characteristics of the study population according to the surgical procedure.

	AGV (*n* = 24)	PGI (*n* = 28)	*p*-value
Age, years			
Mean (SD)	48.3 (17.7)	47.6 (16.8)	0.88^a^
Gender, n (%)			
Male	12 (50.0)	17 (60.7)	0.44^b^
Female	12 (50.0)	11 (39.3)	
Ethnicity, n (%)			
North European	12 (50.0)	21 (75.0)	< 0.01^c^
African	1 (4.2)	3 (10.7)	
South Asian	1 (4.2)	1 (3.6)	
Middle East	2 (8.3)	1 (3.6)	
South European	0 (0.0)	2 (7.1)	
Non available	8 (33.3)	0 (0.0)	
Laterality, n (%)			
Right	7 (29.2)	13 (46.4)	0.20^b^
Left	17 (70.8)	15 (53.6)	
Diabetes, n (%)			
Yes	4 (16.7)	3 (10.7)	0.69^d^
No	20 (83.3)	25 (89.3)	
Diagnosis, n (%)			
Primary open angle glaucoma	2 (8.3)	5 (17.9)	0.35^c^
Secondary open angle glaucoma	13 (54.2)	17 (60.7)	
Neovascular glaucoma	5 (20.8)	2 (7.1)	
Anterior segment developmental anomaly glaucoma	3 (12.5)	4 (14.3)	
Primary angles closure glaucoma	1 (4.2)	0 (0.0)	
Lens status, n (%)			
Pseudophakia	17 (70.8)	17 (60.7)	0.18^c^
Phakia	4 (16.4)	10 (35.7)	
Aphakia	3 (12.5)	1 (3.6)	
Previous intraocular surgery (other than cataract surgery and glaucoma surgery, n (%)			
No	14 (58.3)	18 (64.3)	0.23^c^
Pars plana vitrectomy	8 (33.3)	5 (17.9)	
Penetrating keratoplasty	2 (8.4)	3 (10.7)	
Descemet membrane endothelial keratoplasty	0 (0.0)	1 (3.6)	
Laser-in-situ-Keratomileusis	0 (0.0)	1 (3.6)	
Previous glaucoma laser, n (%)			
Yes	4 (16.7)	1 (3.7)	0.18^d^
No	20 (83.3)	26 (96.3)	
Previous glaucoma surgery, n (%)			
Yes	22 (91.7)	21 (75.0)	0.15^d^
Trabelectomy	(19)	(23)	
Trabeculotomy	(0)	(5)	
Combined trabeculotomy and trabeculectomy	(5)	(5)	
Glaucoma drainage	(2)	(0)	
XEN-stent	(2)	(9)	
Canaloplasty	(2)	(9)	
Open revision	(10)	(5)	
Goniotrepanation	(5)	(0)	
iStent	(5)	(2)	
Ex-PRESS Shunt	(2)	(0)	
Preserflo	(0)	(2)	
No	2 (8.3)	7 (25.0)	
Previous cyclodestructive procedure, n (%)			
Yes	17 (70.8)	14 (50.0)	0.18^b^
No	7 (29.2)	14 (50.0)	
Number of previous glaucoma surgeries			
Median (IQR)	2.0 (2.0)	2.0 (3.0)	0.27^e^
Visual acuity, logMAR			
Median (IQR)	0.9 (1.7)	0.56 (1.35)	0.37^e^
Preoperative IOP, mm Hg			
Median (IQR)	29.5 (12)	34.0 (11)	0.797^e^
Number of classes of intraocular			
Pressure-lowering medications			
Median (IQR)	3.5 (1.0)	3.0 (2.0)	0.40^e^
Oral acetazolamide, n (%)			
Yes	15 (62.5)	16 (57.1)	0.70^b^
No	9 (37.5)	12 (42.9)	
Patch, n (%)			
Tutopatch^®^ bovine pericardium	3 (12.5)	17 (60.7)	< 0.01^c^
Tutoplast^®^ Fascia lata	3 (12.5)	2 (7.1)	
Scleral flap	18 (75.0)	8 (28.6)	
Only scleral tunnel	0 (0)	1 (3.6)	

^a^T-test. ^b^Chi-squared test. ^c^Likelihood Ratio. ^d^Fisher’s Exact Test. ^e^Mann-Whitney test.

### Primary outcomes

#### Intraocular pressure reduction

**Fig. 1:**
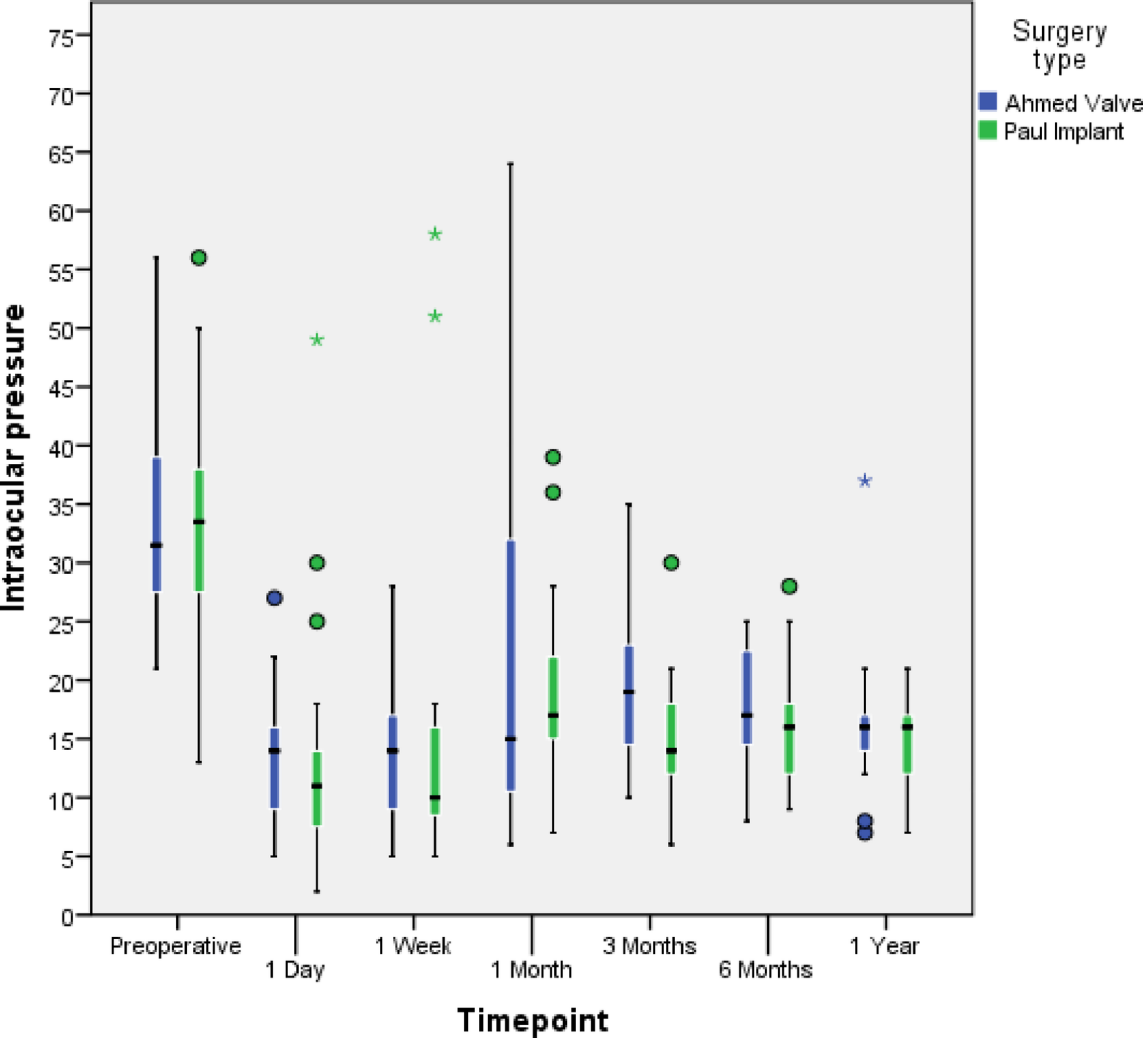
Median IOP values (horizontal black line) and percentiles (boxes-quartiles = 25/75; whiskers = 5/95) given preoperatively and for a follow-up of 1 year. Single outliers are shown by circles and extreme outliers by asterisks.

The median preoperative IOP (IQR) decreased from 29.5mmHg (21–42) to 16.0mmHg (7–37) at the one-year follow-up in the AGV group (*p* < 0.01, Wilcoxon signed-rank test). In the PGI group, it decreased from 34.0 mmHg (13–56) to 16.0 mmHg (7–21) (*p* < 0.01, Wilcoxon signed-rank test). Figure 1 represents the change in IOP from the preoperative value to the one-year follow-up. There were no statistically significant differences between the two groups at any time point (p-values: 0.30, 0.37, 0.69, 0.09, 0.35, 0.35 at 24 h, 1 week, 1 month, 3 months, 6 months, and 1 year postoperatively, respectively; Mann-Whitney Test).

#### Antiglaucoma medication reduction

**Fig. 2:**
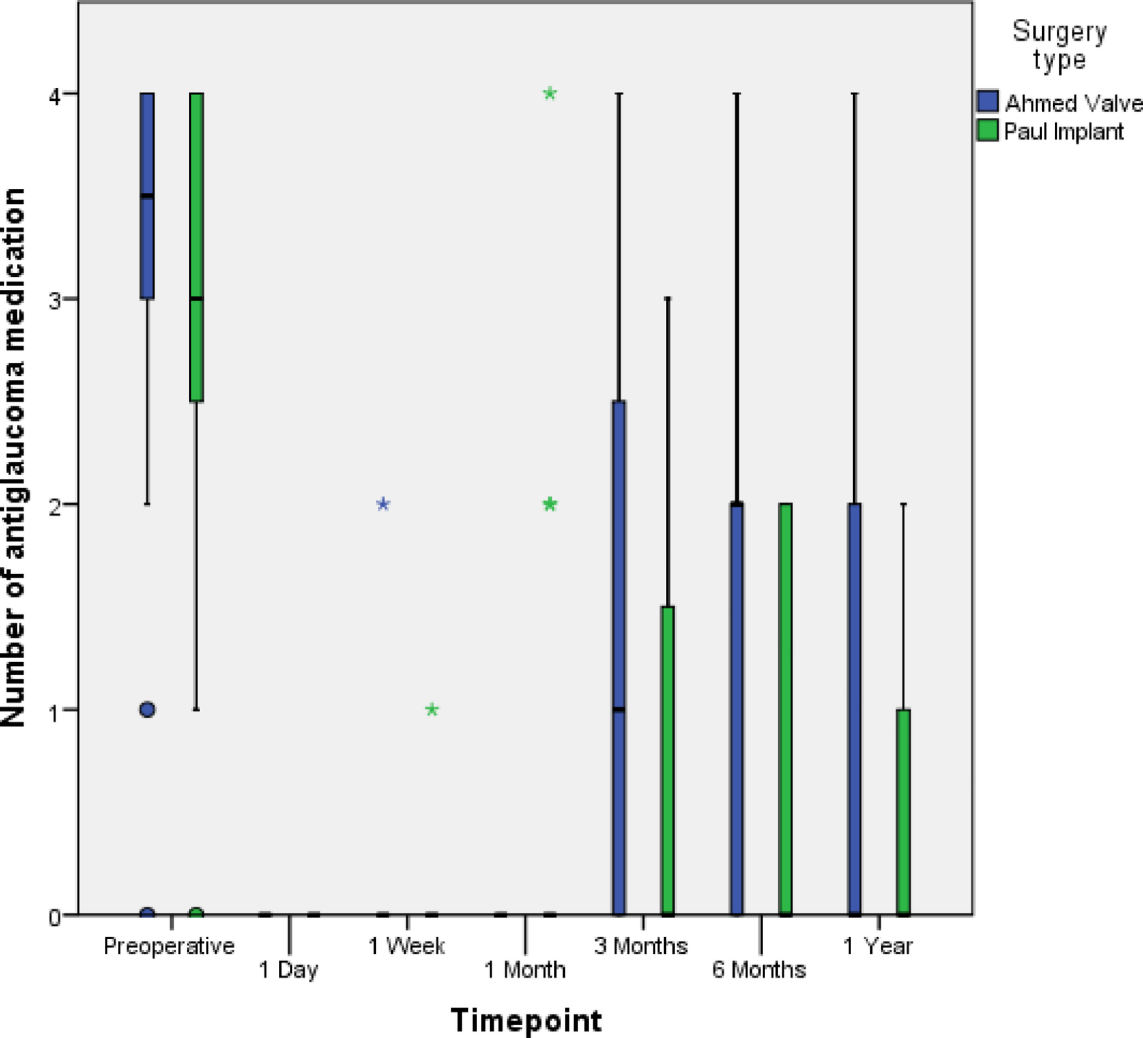
Median number of glaucoma medications (horizontal black line) and percentiles (boxes-quartiles = 25/75; whiskers = 5/95) given preoperatively and for a follow-up of 1 year. Single outliers are shown by circles and extreme outliers by asterisks.

The median (IQR) number of classes of intraocular pressure-lowering medications was 3.5 (0–4) in the AGV group and 3.0 (0–5) in the PGI group at the baseline, and it decreased to 0 (0–4) (*p* < 0.01, Wilcoxon signed-rank test) and 0 (0–3) (*p* < 0.01, Wilcoxon signed-rank test) at one-year follow-up, respectively. There were no statistically significant differences in the number of glaucoma medications between the two groups at any time point (p-values: 1.00, 0.83, 0.09, 0.27, 0.06, 0.33 at 24 h, 1 week, 1 month, 3 months, 6 months, and 1 year postoperatively, respectively; Mann-Whitney Test). See (Fig. 2). The use of oral acetazolamide was reduced from 15 out of 24 patients (62.5%) to 1 out of 18 patients (5.6%) in the AGV group, and from 16 out of 28 patients (57.1%) to 0 out of 22 patients (0%) in the PGI group.

### Secondary outcomes

#### Failure and success

**Fig. 3:**
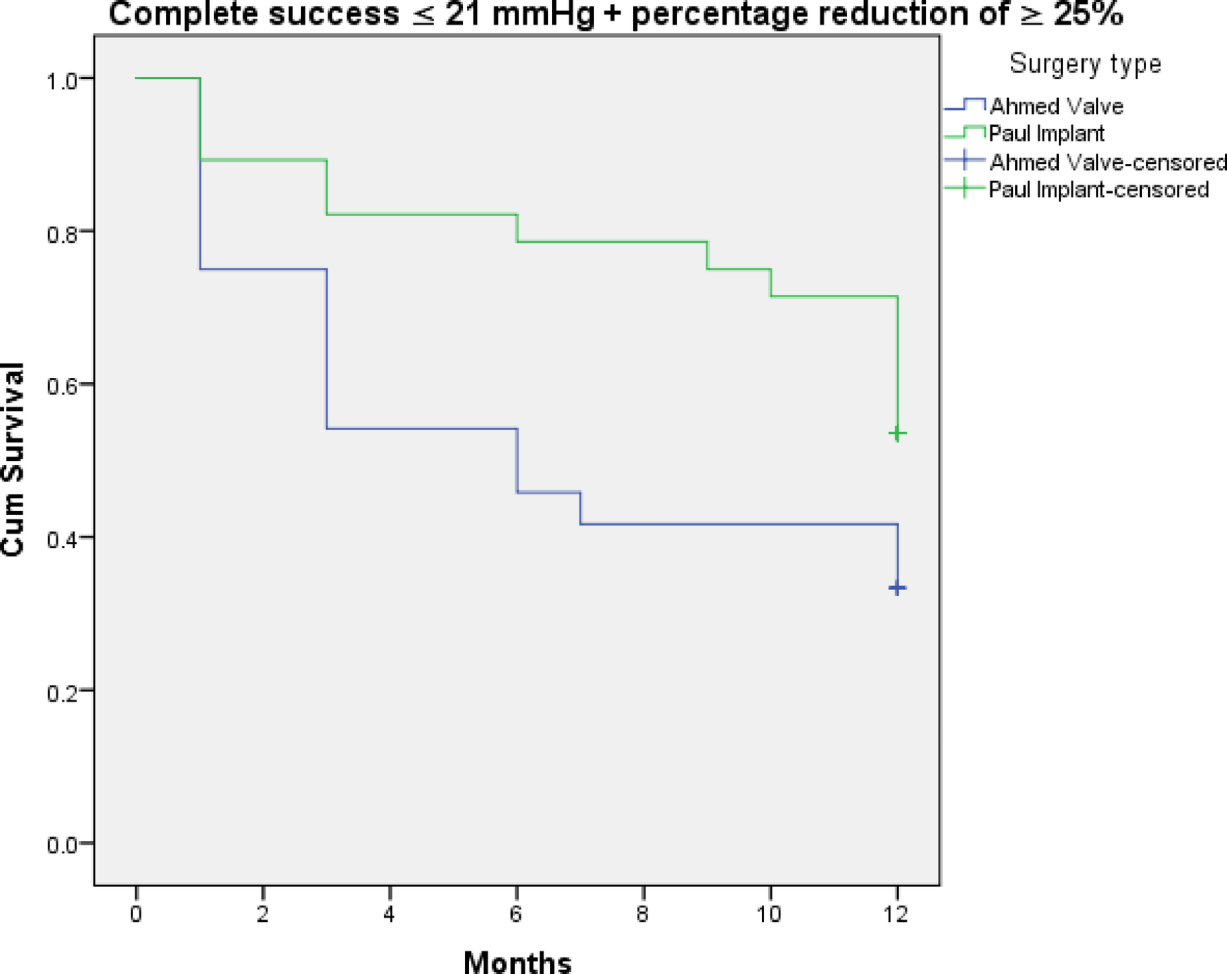
Kaplan-Meier survival curves over 1-year postoperatively for complete success at ≤ 21 mmHg with a reduction of > 25%.

**Fig. 4:**
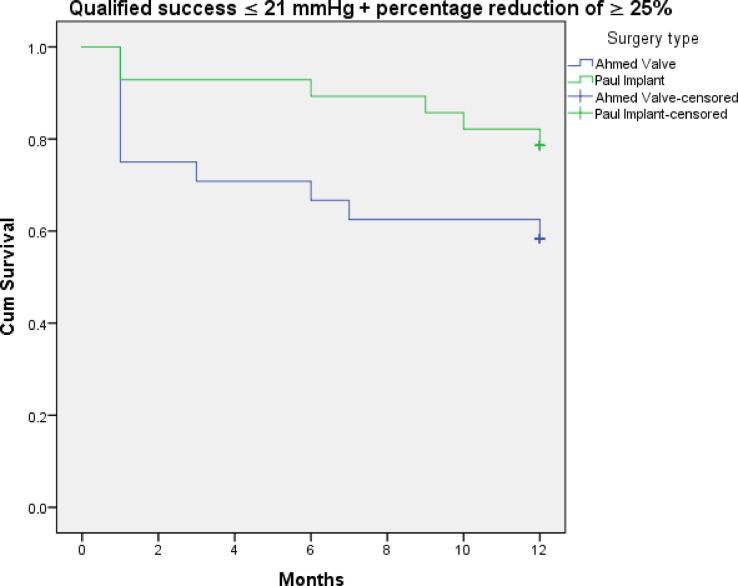
Kaplan-Meier survival curves over 1-year postoperatively for qualified success at ≤ 21 mmHg with a reduction of > 25%.

**Fig. 5:**
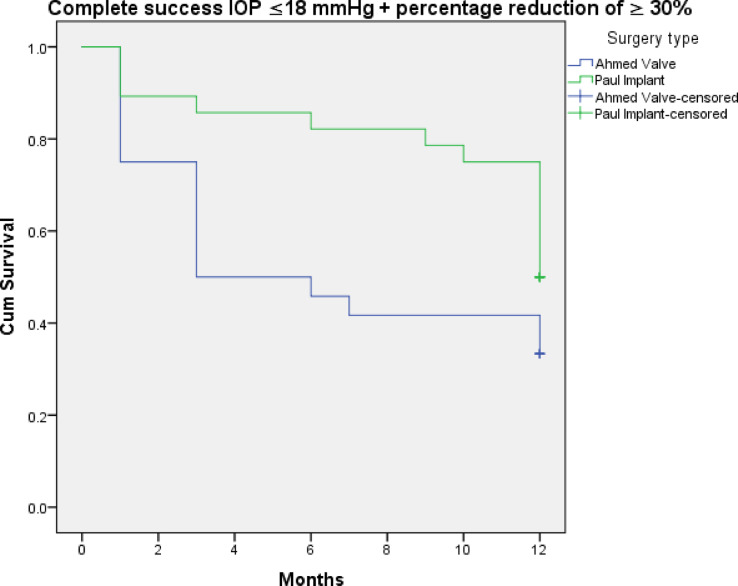
Kaplan-Meier survival curves over 1-year postoperatively for complete success at ≤ 18 mmHg with a reduction of > 30%.

**Fig. 6:**
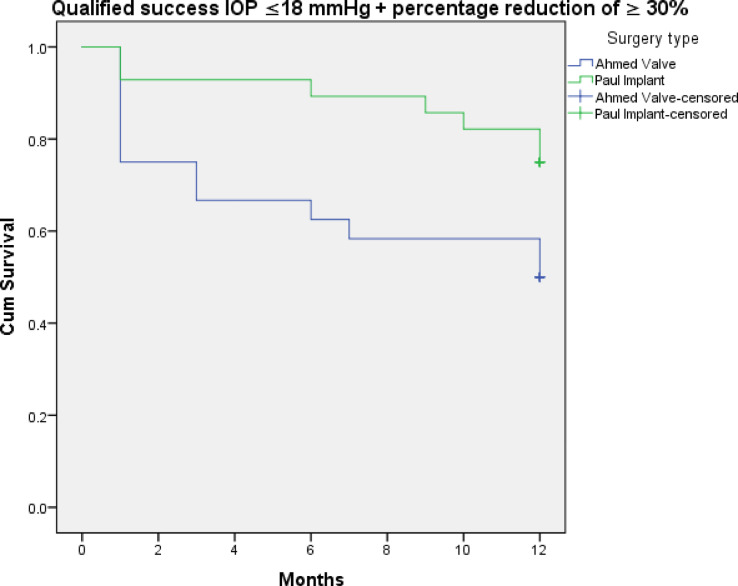
Kaplan-Meier survival curves over 1-year postoperatively for qualified success at ≤ 18 mmHg with a reduction of > 30%.

**Fig. 7:**
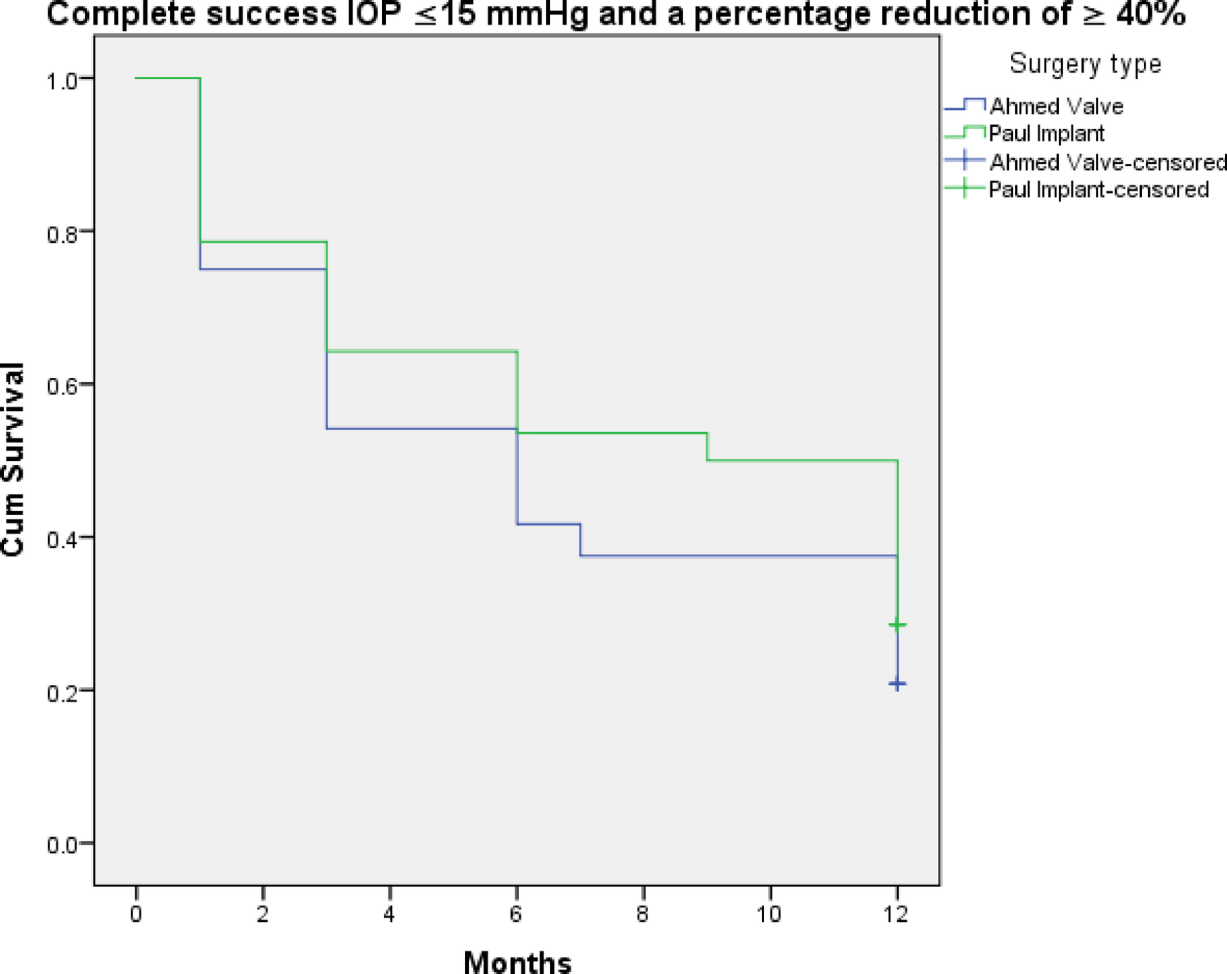
Kaplan-Meier survival curves over 1-year postoperatively for complete success at ≤ 15 mmHg with a reduction of > 40%.

**Fig. 8:**
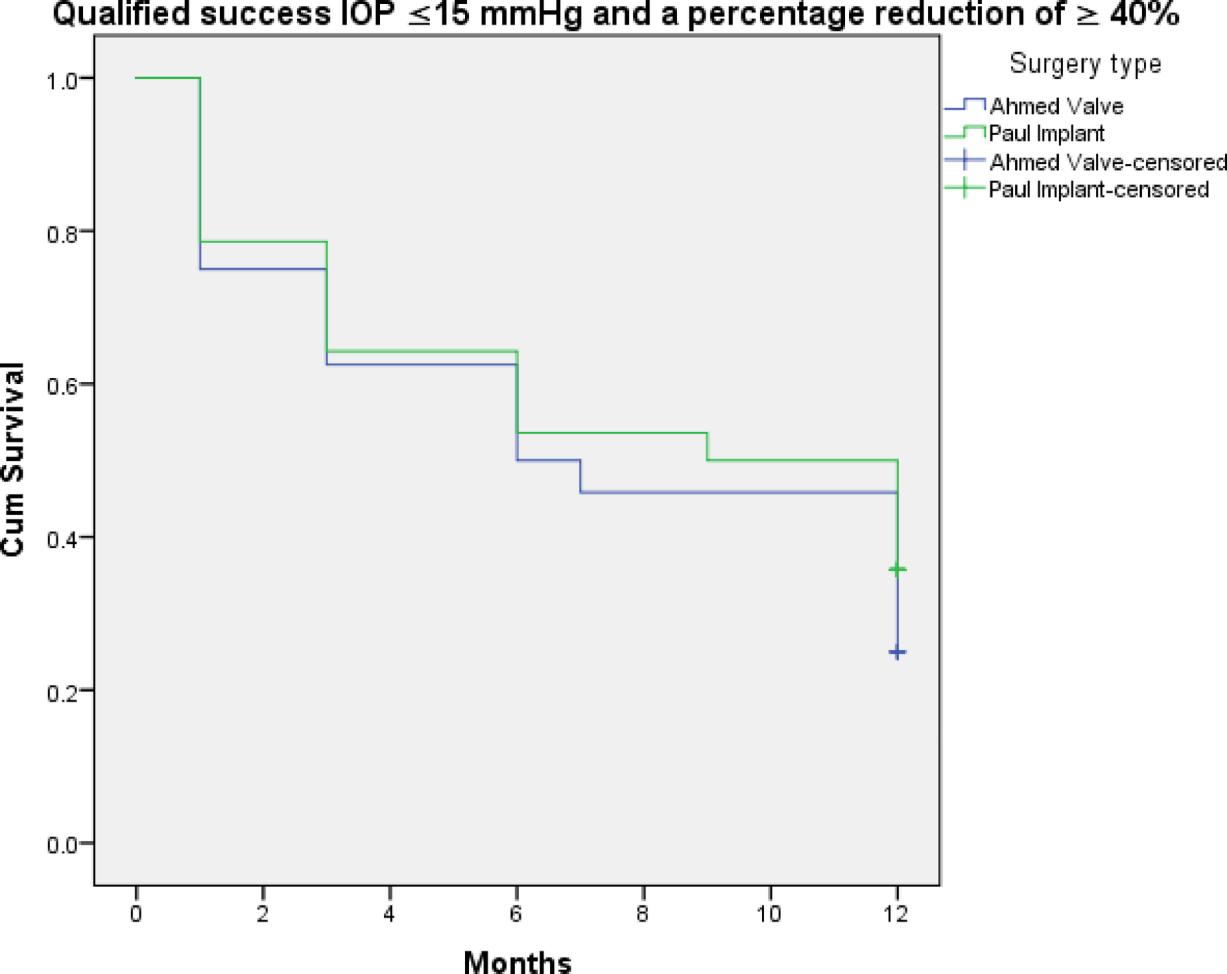
Kaplan-Meier survival curves over 1-year postoperatively for qualified success at ≤ 15 mmHg with a reduction of > 40%.

Figures 3, 4, 5, 6, 7 and 8 shows the Kaplan-Meier survival curve over the 1-year follow-up period for the different IOP targets. There were no statistically significant differences between the curves for complete and qualified success for IOP targets 1 and 3 between both groups (Fig. 3: *p* = 0.062, Fig. 4: *p* = 0.095, Fig. 7: *p* = 0.411, and Fig. 8: *p* = 0.508, respectively, Log-Rank Test). Survival was also not statistically significant for complete success at IOP target 2 ( Fig. 5: *p* = 0.069), but there was a higher probability of survival for qualified success at IOP target 2 in the PGI group (Fig. 6: *p* = 0.045).

Reasons for failure in the AGV group included 8 open revisions due device obstruction and uncontrolled intraocular pressure (IOP) and one patient whose IOP was 37 mmHg but could be managed medically in the following month.

In the PGI group, one patient with open-angle glaucoma required anterior chamber washing two days post-surgery due to an IOP of 49 mmHg, up from a preoperative value of 33 mmHg, which could not be controlled medically. Another patient with active uveitic glaucoma experienced an IOP peak of 58 mmHg in the first week, up from a preoperative value of 36 mmHg, which was medically managed down to 26 mmHg but had recurrent episodes of hyphema and vitreous haemorrhage requiring open revision. Additionally, a patient with neovascular glaucoma had an IOP peak of 51 mmHg in the first week that did not respond to medical therapy, necessitating cyclocryocoagulation. The other two patients in the PGI group had encapsulated devices requiring open revision.

#### Postoperative visual acuity

The median preoperative visual acuity (IQR) was stable from 0.95 (1.70) to 1.0 (2.1) logMAR at the one-year follow-up in the AGV group (*p* = 0.15, Wilcoxon signed-rank test). In the PGI group, it was also stable from 0.56 (1.35) to 0.55 (2.08) logMAR (*p* = 0.78, Wilcoxon signed-rank test).

#### Intraoperative and postoperative complications

The frequency of intraoperative and postoperative complications are shown in (Table 2). There was no statistically significant difference in intraoperative and postoperative complications between the two groups, except for device obstruction and device exposure. Device obstruction was significantly more frequent in the AGV group (*p* = 0.005, Fisher’s exact test), while tube exposure occurred more frequently in the PGI group (*p* = 0.036, Chi-squared test). However, these associations could not be confirmed through logistic regression due to high uncertainty in the estimates. Bootstrapping revealed large standard errors and wide confidence intervals for the coefficients (0.137–10.458), likely due to the small sample size, which limits the reliability of the logistic regression model. Logistic regression analysis between the use of a patch and the occurrence of tube exposure revealed no statistically significant difference (*p* = 0.31, Wald test).

**Table 2:** Adverse events.

	AGV *n* (%)	PGI *n* (%)	*p*-value
Intraoperative complications	4/24 (16.7)	1/28 (3.6)	0.17^a^
Scleral damage^1^	1/4	1/1	
Muscle damage^2^	1/4		
Hyphema	1/4		
Conjunctival tear	1/4		
Postoperative complications	17/24 (70.8)	17/28 (60.7)	0.56^b^
Early complications^3^	13/24 (54.2)	11/28 (50.0)	0.28^b^
Late complications^4^	11/24 (45.8)	14/28 (50.0)	0.76^b^
Numerical Hypotony (IOP < 6mmHg)			
Early	3/24 (12.5)	4/28 (14.3)	0.87^a^
Late	1/24 (4.2)	2/28 (7.1)	
Choroidal detachment			
Early	0/24 (0)	1/28 (3.6)	0.26^a^
Late	0/24 (0)	0/28 (0)	
Severe anterior chamber shallowing			
Early	1/24 (4.2)	0/28 (0)	0.21^a^
Late	0/24 (0)	0/28 (0)	
Visible hyphema			
Early	2/24 (8.3)	2/28 (7.1)	0.87^a^
Late	0/24 (0)	0/28 (0)	
IOP peak			
Early	1/24 (4.2)	4/28 (14.3)	0.38^a^
Late	1/24 (4.2)	2/28 (7.1)	
Cataract progression			
Early	0/4 (0)	1/10 (10)	1.00^a^
Late	0/4 (0)	0/10 (0)	
Persistent corneal erosion			
Early	0/24 (0)	1/28 (3.6)	0.26^a^
Late	0/24 (0)	0/28 (0)	
Corneal decompensation			
Early	2/24 (8.3)	1/28 (3.6)	0.53^a^
Late	1/24 (4.2)	3/28 (10.7)	
Persistent uveitis	2/24 (8.3)	2/28 (7.1)	1.00^a^
Blinding visual acuity loss			
Early	1/24 (4.2)	0/28 (0)	0.46^a^
Late	0/24 (0)	0/28 (0)	
Choroidal Haemorrhage			
Early	1/24 (4.2)	0/28 (0)	0.46^a^
Late	0/24 (0)	0/28 (0)	
Macular oedema			
Early	2/24 (8.3)	3/28 (10.7)	1.00^a^
Late	0/24 (0)	0/28 (0)	
Retinal detachment			
Early	1/24 (4.2)	1/28 (3.6)	1.00^a^
Late	0/24 (0)	0/28 (0)	
Vitreous Haemorrhage			
Early	2/24 (8.3)	1/28 (3.6)	0.59^a^
Late	0/24 (0)	0/28 (0)	
Diplopia			
Early	0/24 (0)	1/28 (3.6)	1.00^a^
Late	0/24 (0)	0/28 (0)	
Device obstruction and encapsulation			
Early	6/24(25.0)	0/28 (0)	0.01^a^
Late	2/24 (8.3)	2/28 (7.1)	
Device migration^5^			
Early	1/24 (4.2)	0/28 (0)	0.46^a^
Late	0/24 (0)	0/28 (0)	
Device malposition^6^			
Early	0/24 (0)	1/28 (3.6)	1.00^a^
Late	0/24 (0)	0/28 (0)	
Fibrous ingrowth			
Early	0/24 (0)	1/28 (3.6)	1.00^a^
Late	0/24 (0)	0/28 (0)	
Device exposure			
Early	1/24(4.2)	0/28 (0)	0.04^a^
Late	0/24 (0)	4/28 (14.3)	
Need of reintervention			
Yes	10/24 (41.7)	9/28 (32.1)	0.57^a^
No	0/24 (0)	0/28 (0)	

^a^Fisher’s Exact Test. ^b^Chi-squared test.

^1^Unintended penetration of the sclera in an improper location, requiring suturing due to the use of surgical instruments.

^2^Accidental tearing of muscle tissue during the preparation of the conjunctiva with surgical instruments.

^3^Early complications refer to those that occurred within the first three months after the operation.

^4^Late complications refer to those that occurred after the first three months after the operation.

^5^The positioning of a glaucoma device or its components in an undesired location relative to its intended position, caused by a postoperative shift from its original position.

^6^The positioning of a glaucoma implant or any of its components in an undesired location relative to its intended position, not due to the migration of a previously well-positioned implant.

Regarding the patients with corneal decompensation, in the AGV group, one had a history of penetrating keratoplasty, another had a history of ICE Syndrome, and a third had a diagnosis of congenital glaucoma with seven previous glaucoma surgeries. In the PGI group, one patient had a diagnosis of congenital glaucoma with four previous glaucoma surgeries, another had uveitic glaucoma with a history of two previous cyclodestructive procedures, one had a previous Descemet Membrane Endothelial Keratoplasty (DMEK), and another had a history of penetrating keratoplasty.

In the AGV group, reintervention was required in eight cases due to uncontrolled intraocular pressure, which necessitated open revisions. Additionally, one case required intervention for tube exposure through the conjunctiva, and another involved an injection of Healon into the anterior chamber to address choroidal detachment associated with an intraocular pressure of 10 mmHg. In the PGI group, four patients required reintervention due to tube exposure. Additionally, one patient needed cyclocryocoagulation, three patients required open revisions due to elevated intraocular pressure, and one patient needed tube shortening in the anterior chamber following an episode of vasculitis and uveitis with iris bombe, which caused the tube to be in direct contact with the iris. Only interventions to control intraocular pressure or the need for removal of the implant were considered failures.

The ripcord (Prolene 6 − 0) was removed in 12 patients (42.9%). The mean intraocular pressure prior to extraction was 31.1 mmHg (SD 7.44), and the mean time to extraction was 253.1 days (SD 149.4) after surgery. The mean intraocular pressure at the end of the follow-up in these patients was 11.2 mmHg (SD 4.21). There were two cases of hypotony; one occurred in a patient with posterior uveitis, who did not recover from the hypotony (2 mmHg) and lost vision to light perception due to persistent inflammation. The other case recovered from 5 to 7 mmHg without clinical signs of hypotony.

## Discussion

This study analysed one-year outcomes for 24 and 28 adult patients, predominantly diagnosed with secondary glaucoma, who received either an Ahmed Glaucoma Valve or a Paul Glaucoma Implant at the University Medical Centre Mainz. At the time of this analysis, only one study had been published comparing these two glaucoma drainage devices^16^.

Our study demonstrated that the AGV group experienced a significant reduction in median preoperative IOP (IQR), which decreased from 29.5 mmHg (21–42) to 16.0 mmHg (7–37) at the one-year follow-up (*p* < 0.01, Wilcoxon signed-rank test). Similarly, the PGI group showed a significant decrease from 34.0 mmHg (13–56) to 16.0 mmHg (7–21) (*p* < 0.01, Wilcoxon signed-rank test). Additionally, the median number of postoperative medications significantly decreased from 3.5 (IQR: 0–4) to 0 (IQR: 0–4) in the AGV group (*p* < 0.01) and from 3.0 (IQR: 0–5) to 0 (0–3) in the PGI group (*p* < 0.01). There were no statistically significant differences between the two groups as observed by the study published by Karapapak et al.^16^.

The decrease in IOP observed by Karapapak et al. at the one-year follow-up was slightly greater than that observed in our study for both groups: AGV (62.09% vs. 44.7%) and PGI (66.25% vs. 50.6%)^16^. However, the mean number of glaucoma medications used in their study was higher, which could account for the differences observed between their results and ours. Karapapak et al. compared the results of AGV and PGI in 18 patients per group, all diagnosed with secondary glaucoma due to silicone oil^16^. In their study, AGV reduced the IOP from 39.3 ± 10 mmHg to 14.9 ± 4.2 mmHg, while PGI reduced the mean IOP from 40 ± 13 mmHg to 13.5 ± 2.2 mmHg^16^. Additionally, AGV decreased the mean number of medications from 4 ± 0 to 1.9 ± 1.8, and PGI from 3.8 ± 0.4 to 1.7 ± 1.3^16^.

The criteria used by Karapapak et al. for surgical success at the end of the 12-month follow-up period included patients with IOP ≤ 21 mmHg or ≥ 6 mmHg and no loss of light perception^16^. They did not consider a percentage reduction in IOP as necessary for surgical success, nor did they count additional surgical procedures due to elevated IOP as failures^16^. In fact, six patients in the AGV group required additional surgery, and the final reported surgical success was 16/18 (89%) in the AGV group and 17/18 (94%) in the PGI group^16^. If we also include patients who required surgery to control IOP in our analysis, our surgical success rate would increase from 62.5 to 83.3% in the AGV group and from 75.0 to 92.9% in the PGI group.

Kurapapak et al. reported complications in 44.4% of AGV patients and 22.2% of PGI patients, with no statistically significant difference between the groups^16^. Our complication rate was higher, at 70% in the AGV group and 60.7% in the PGI group, but also showed no significant difference between the groups. Our cohort includes various types of secondary glaucoma, such as neovascular glaucoma and adult congenital glaucoma, both of which are well-known for their challenging management and high rates of postoperative complications. They reported hyphema in 3 out of 18 PGI patients (16.7%) (compared to our 7.1% patients) and one case of pupillary membrane (5.6%); in the AGV group, they found hyphema in 11.1% patients (compared to our 8.3%) and six cases of acute hypotony (33.3%) (compared to our 16.6%)^16^. They did not report any incidence of hypotony in the PGI group, while we reported 21.4%^16^. Additionally, they did not observe any tube exposure cases, despite using a pericardium patch in every patient, as we did^16^. They also found more bleb encapsulation in the AGV group compared to the PGI group^16^. They removed the ripcord in 14 out of 18 patients (77.8%) with a mean time of 30.1 ± 14.8 days, compared to our 12 out of 28 patients (42.9%) with a mean extraction time of 253.1 ± 149.4 days^16^. No patients in their PGI group required additional surgery, whereas nine patients in our cohort did. Additionally, six patients in their AGV group required further surgery, compared to ten patients in our cohort^16^.

A potential explanation for these differences is the duration of glaucoma evolution. Patients in Karapapak’s study were diagnosed with glaucoma secondary to silicone oil, and those with a history of glaucoma prior to vitreoretinal surgery were excluded^16^. This suggests a shorter disease duration in their cohort compared to ours, and possibly fewer prior glaucoma procedures than our patients. Although Karapapak et al. did not report the mean number of glaucoma surgeries per group, they noted that all 18 patients in the PGI group had undergone at least one glaucoma surgery, compared to 13 out of 18 patients in the AGV group^16^. In our study, 22 out of 24 patients (91.7%) in the AGV group and 21 out of 28 patients (75.0%) in the PGI group had undergone previous glaucoma surgery. Our mean number of previous glaucoma surgeries was 2.6 ± 1.9 in the AGV group and 1.9 ± 1.6 in the PGI group.

Berteloot et al. conducted a comparative study of PGI and BGI at the one-year mark, excluding patients with neovascular glaucoma^18^. In their study, 43% of the patients in the PGI group had POAG, compared to 33% in the BGI group^18^. In contrast, our cohort had POAG in 8.3% of the patients in the AGV group and 17.9% in the PGI group. Beteloot et al. reported results for 23 patients with PGI and 27 patients with BGI, finding that the BGI group had a statistically significant lower IOP at the 12-month follow-up^18^. The IOP decreased from 23.7 ± 6.9 to 13.1 ± 2.9 mmHg in the PGI group, and from 26 ± 7.3 to 10.4 ± 4.9 mmHg in the BGI group^18^. The percentage reduction in IOP for the PGI group was 44.6%, closely matching our result of 50.6%^18^. Both groups showed no difference in terms of medication use at the 12-month follow-up, with the PGI group reducing medication from 2.7 ± 1.1 to 1.41 ± 1.4, though this end value was greater than ours^18^. Their success rates, defined as an IOP between 6 and 18 mmHg and at least a 20% reduction from baseline, were similar for both groups: 91% for the PGI and 89% for the BGI. In the PGI group, two cases were considered failures: one due to severe hypotony on day 3 requiring intervention, and another with late hypotony after ripcord removal^18^. In the BGI group, three cases were deemed failures: one requiring tube revision due to posterior displacement, another needing tube flushing and needling, and a third presenting with loss of light perception after ripcord removal due to vitreous bleeding and corneal staining^18^. There were no significant differences in complication rates between the two groups^18^. The PGI group experienced early complications in 21.7% of cases and late complications in 65.2%, but they did not record the number of patients with numerical hypotony^18^. No tube exposure was observed in the PGI group, although three patients developed encapsulated blebs^18^. Ripcord removal occurred in 29 of the 23 PGI patients (87.0%), approximately double the proportion in our cohort, which may explain the lower IOP observed compared to our results^18^.

Despite the similarities between the PGI and BGI, such as their valveless systems and comparable endplate surface areas, the IOP outcomes for the PGI tend to resemble those of the Ahmed Glaucoma Valve^16,18^. This difference may lie in the exposure of the bleb to aqueous humor during the early phases of bleb formation. Aqueous humor contains various growth factors that induce fibroblasts to synthesize collagen and transform into myofibroblasts^20^. Reduced exposure of the bleb to aqueous humor may decrease the fibrosis of the capsule^20^. This could also explain why the Ahmed Glaucoma Valve produced more encapsulated blebs that require surgical intervention in our study, as the bleb in this case is more exposed to aqueous humor from the beginning of wound healing. Conversely, we did not find a significant percentage of patients with peak intraocular pressure or hypotony with these devices.

Our results align with those reported in studies of PGI in adults^12–18,21–23^ and fall within the range of IOP and the number of antiglaucoma medications reduction published in studies with the AGV^7,10,24,25^.

The heterogeneity in the definitions of surgical success makes comparing studies very challenging. Studies that employ less criteria tend to report higher rates of success^12,13,15,22^. Our definition of success is strict, yet our success rates are close to the range reported by other studies.

The rates of ripcord removal range from 42 to 87% within 1 to 5 months after surgery^16,18,21–23^. Not all surgeons used an intraluminal stent to prevent hypotony with PGI. Studies using an intraluminal ripcord reported varying frequencies of hypotony ranging from 0 to 17.1%^16,18,21–23^, though they considered hypotony only when accompanied by clinical signs. Although we observed a numerical hypotony in 6 out of 28 patients (21.42%), only one patient (3.6%) showed choroidal detachment as a clinical sign of hypotony, which resolved spontaneously. None of the patients in our PGI group required intervention due to hypotony.

Regarding tube exposure, while Weber et al.^21^suggested a potential association with the pericardial patch, our logistic regression analysis found no statistically significant correlation between tube exposure and the type of patch, regardless of the GDD used. However, there was a trend indicating higher exposure rates with pericardium grafts compared to fascia lata or scleral flaps. In our study, most AGV devices were covered by scleral flaps, whereas pericardium patches were used for the PGI. Studies using pericardium grafts in PGI reported tube exposure ranging from 0 to 16.1%^12,16–18,21,23^.

Our study has several limitations: the small sample size, potential bias due to its retrospective nature, and a limited 12-month follow-up. As a well-recognized glaucoma center, we may mainly report data from patients who return with complications. The heterogeneous patient population, especially regarding ethnic background, and the possibility that non-returning patients had either good outcomes or sought care elsewhere further limit our findings. Longer-term, prospective studies with larger sample sizes are needed to demonstrate the superiority or equivalence of these two procedures.

## Conclusion

The AGV has an established track record and a proven safety profile. The PGI demonstrates comparable efficacy and safety, with most complications being minor. However, the PGI showed superior performance in terms of qualified surgical success, achieving an IOP ≤ 18 mmHg and a 30% reduction from baseline IOP at the one-year follow-up.

## Data Availability

Data is available upon request from the corresponding author.
